# CD56^bright^ NK cells are negatively associated with antibody response to vaccination in people with multiple sclerosis on B‐cell‐depleting therapy

**DOI:** 10.1002/cti2.70079

**Published:** 2026-02-10

**Authors:** Griffith B Perkins, Christopher M Hope, Cheng Sheng Chai, Matthew J Tunbridge, Sebastian Sterling, Kevin Webb, Joey Yap, Arthur Eng Lip Yeow, Makutiro G Masavuli, Jacqueline Scaffidi, James D Zuiani, Anouschka Akerman, Anupriya Aggarwal, Vanessa Milogiannakis, Matthew B Roberts, Plinio R Hurtado, Stuart Turville, Branka Grubor‐Bauk, Simon C Barry, Janakan Ravindran, Patrick Toby Coates, Pravin Hissaria

**Affiliations:** ^1^ Central and Northern Adelaide Renal and Transplantation Service Royal Adelaide Hospital Adelaide SA Australia; ^2^ School of Medicine Adelaide University Adelaide SA Australia; ^3^ Immunology Directorate, SA Pathology Adelaide SA Australia; ^4^ Molecular Immunology, Robinson Research Institute Adelaide University Adelaide SA Australia; ^5^ Department of Paediatric Medicine Women's and Children's Hospital North Adelaide SA Australia; ^6^ Central Adelaide Local Health Network Adelaide SA Australia; ^7^ Department of Neurology Royal Adelaide Hospital Adelaide SA Australia; ^8^ Viral Immunology Group, Basil Hetzel Institute for Translational Health Research Adelaide University Woodville SA Australia; ^9^ Kirby Institute University of New South Wales Sydney NSW Australia; ^10^ Infectious Diseases Royal Adelaide Hospital Adelaide SA Australia; ^11^ Department of Immunology Royal Adelaide Hospital Adelaide SA Australia

**Keywords:** B‐cell depletion, CD56^bright^ NK cells, multiple sclerosis, vaccination, virus neutralisation

## Abstract

**Objectives:**

We sought to identify determinants of vaccine response in people with multiple sclerosis (pwMS) receiving B‐cell‐depleting therapies.

**Methods:**

This was a prospective single‐centre cohort study (ACTRN12623001249640). Peripheral blood samples were collected from pwMS receiving ocrelizumab (*n* = 38) before and after a third dose of COVID‐19 mRNA vaccine. Immunogenicity was measured by T‐cell IFN‐γ ELISpot, antibody titres and live virus neutralisation. Humoral immunity was benchmarked against pwMS receiving natalizumab (*n* = 15), and against a correlate of real‐world protection (50% reduction in incidence of infection). The peripheral immune phenotype was assessed by high‐parameter flow cytometry and tested for association with vaccine response.

**Results:**

CD20^+^ T cells, natural killer (NK) cells and B cells were lower in pwMS receiving ocrelizumab, while CD27^+^CD38^+^ T‐cell and CD8^+^ NK cell frequencies were elevated relative to natalizumab. Following a third dose, 51% of pwMS on ocrelizumab were seropositive for SARS‐CoV‐2 receptor‐binding domain Ig, and 25% and 14% met the threshold for effective neutralisation of live ancestral and omicron BA.5 virus, respectively. B‐cell frequency at the time of vaccination, but not time since ocrelizumab infusion, positively correlated with antibody response. Immunomodulatory CD56^bright^ NK cells were negatively associated with antibody response. CD3^−^CD20^+^ B cells (% of lymphocytes) and CD56^bright^ NK cells (% of NK cells) were associated with effective virus neutralisation in prior non‐responders.

**Conclusion:**

Time since ocrelizumab infusion was not associated with protective vaccination. Evaluation of B‐cell and CD56^bright^ NK cell frequencies may provide a personalised strategy to stratify pwMS for vaccination and prophylaxis.

## Graphical abstract

Antibody response to vaccination in people with multiple sclerosis receiving B‐cell depletion therapy is associated with B‐cell frequency but not time since B‐cell depletion. Immunoregulatory CD56^bright^ NK cells may negatively impact antibody responses in this group.

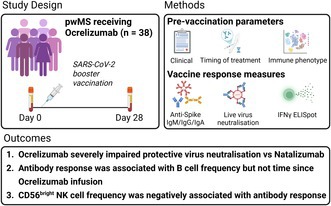

## Introduction

Disease modifying therapies (DMTs) employed in the treatment of relapsing–remitting multiple sclerosis (RRMS) can affect the immune response to vaccination. Common DMTs include the B‐cell‐depleting anti‐CD20 monoclonal antibody, ocrelizumab (Ocrevus) and the anti‐α4‐integrin monoclonal antibody, natalizumab (Tysabri). B‐cell‐depleting therapies (BCDTs) are associated with increased risk of severe COVID‐19 disease^1^, ^2^, ^3^, ^4^, ^5^ and people with multiple sclerosis (pwMS) on BCDT have among the lowest rates of seroconversion to COVID‐19 vaccination of any group.^5^, ^6^, ^7^, ^8^, ^9^, ^10^, ^11^, ^12^ In those who do seroconvert, antibody titres are inferior to healthy individuals. As such, it is difficult to interpret the level of protection afforded these patients (particularly against antibody‐escape variants, such as omicron BA.5), and for clinicians and policy makers to make informed decisions with respect to booster dosing and existing and emerging prophylactic measures. Natalizumab, in contrast, is non‐depleting, and no impairment in vaccine response has been identified for pwMS receiving treatment with natalizumab.^6^, ^8^, ^13^

B‐cell depletion produces significant changes in the immune system, which influence both vaccine response and MS pathogenesis.^14^ Ablation of the B‐cell compartment directly impairs the antibody response, and BCDT infusion intervals (six monthly for ocrelizumab) are optimised to maintain B‐cell depletion. Despite this, up to 50% of pwMS receiving BCDT seroconvert following three COVID‐19 vaccine doses. It remains to be determined whether B‐cell reconstitution alone or in combination with other clinical and immunological factors determines a productive vaccine response. In addition to direct B‐cell depletion, anti‐CD20 monoclonal antibodies target minor CD20‐expressing T‐cell and natural killer (NK) cell populations that have been implicated in MS pathogenesis and produce indirect effects on T‐cell phenotype and function, which has been proposed to mediate some of the beneficial effect of BCDT in MS.^14^, ^15^

Here, we describe cellular and humoral immune responses to a third dose of mRNA‐platform (BNT162b2 Pfizer or mRNA1273 Moderna) COVID‐19 vaccine in pwMS receiving ocrelizumab. Seroconversion rates were compared against pwMS receiving natalizumab, and against a correlate of real‐world protection from infection based on live virus neutralisation of ancestral and omicron BA.5 in order to estimate the level of protection afforded by a third vaccine dose. Finally, we describe phenotypic changes in the peripheral immune system associated with ocrelizumab use and identify potential clinical and phenotypic predictors of response to vaccination.

## Results

Study demographics are presented in Table 1. Thirty‐eight participants receiving ocrelizumab were enrolled in the study and peripheral blood samples were collected immediately prior to, and 4 weeks after, the third vaccine dose. For comparison of immune phenotype and vaccine response, samples were collected from 15 pwMS receiving natalizumab. Median age (44 vs 44 years), sex (68% vs 73% female) and primary COVID‐19 vaccine type (95% vs 100% mRNA‐platform) were similar between treatment groups, and all participants received an mRNA vaccine (BNT162b2 Pfizer or mRNA1273 Moderna) as a third dose. The median time from vaccination to blood sample collection was 28 days (IQR: 28–31) for the ocrelizumab group and 166 days (IQR: 49–191) for the natalizumab group. Five participants in the natalizumab group, and four in the ocrelizumab group, had COVID‐19 prior to the study and were excluded from analyses unless indicated.

**Table 1:** Participant characteristics

	All participants (*N* = 53)	Ocrelizumab (*N* = 38)	Natalizumab (*N* = 15)
Sex			
Female, *N* (%)	37 (69.8)	26 (68.4)	11 (73.3)
Male, *N* (%)	16 (30.2)	12 (31.6)	4 (26.7)
Age, median (IQR), years	44 (35–54)	44 (36–53)	44 (33–54)
MS type			
Relapsing–remitting (%)	53 (100)	38 (100)	15 (100)
Progressive (%)	0 (0)	0 (0)	0 (0)
Days from last ocrelizumab or natalizumab infusion to third vaccine dose, median (IQR)	N/A (N/A)	158 (113–183)	20 (8–24)
First vaccine, *N* (%)			
BNT162b2 (Pfizer)	50 (93.3)	35 (92.1)	15 (100)
mRNA‐1273 (Moderna)	1 (1.9)	1 (2.6)	0 (0)
ChAdOx1 (AstraZeneca)	2 (3.8)	2 (5.3)	0 (0)
Second vaccine, *N* (%)			
BNT162b2 (Pfizer)	51 (96.2)	36 (94.7)	15 (100)
mRNA‐1273 (Moderna)	1 (1.9)	1 (2.6)	0 (0)
ChAdOx1 (AstraZeneca)	1 (1.9)	1 (2.6)	0 (0)
Third vaccine, *N* (%)			
BNT162b2 (Pfizer)	51 (96.2)	38 (100)	13 (86.7)
mRNA‐1273 (Moderna)	2 (3.8)	0 (0)	2 (13.3)
ChAdOx1 (AstraZeneca)	0 (0)	0 (0)	0 (0)
Time between third vaccine dose and sample, median (IQR), days	29 (28–155)	28 (28–31)	166 (49–191)
Contracted COVID prior to study, *N* (%)	9 (17.0)	4 (10.5)	5 (33.3)
Contracted COVID during follow‐up, *N* (%)	16 (30.2)	11 (28.9)	5 (41.7)
Remdesivir, *N* (%)^a^	2 (12.5)	2 (18.2)	0 (0)
Sotrovimab, *N* (%)^a^	3 (18.8)	3 (27.3)	0 (0)
Nirmatrelvir/ritonavir, *N* (%)^a^	2 (12.5)	2 (18.2)	0 (0)
Tixagevimab/cilgavimab, *N* (%)^a^	7 (43.8)	7 (63.6)	0 (0)

^a^ % of COVID positive during follow‐up.

### Booster vaccination improves SARS‐CoV‐2‐specific antibody and T‐cell responses

**Figure 1:**
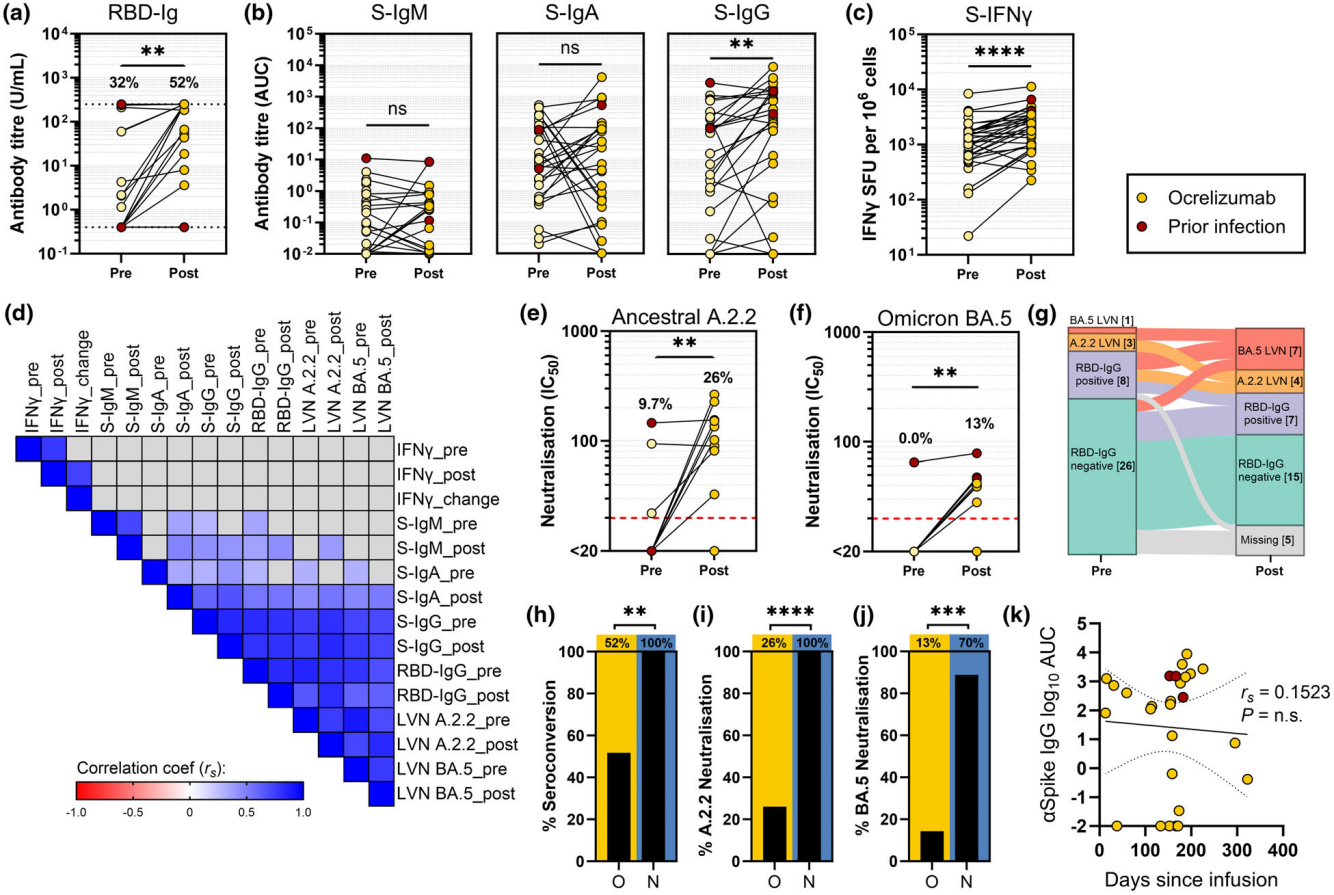
Effect of a third vaccine dose on protective immunity to SARS‐CoV‐2 ancestral and omicron BA.5 variants in people with multiple sclerosis (pwMS) on B‐cell depletion therapy. **(a)** Anti‐SARS‐CoV‐2 receptor‐binding domain Ig (RBD‐Ig) titres. Dashed lines demarcate the upper (250 U/mL) and lower (0.4 U/mL) detection limits of the assay, and percentage seropositivity (> 0.4 U/mL) pre‐ and post‐booster vaccination is indicated for *n* = 31 infection‐naïve participants with paired pre‐ and post‐vaccination data. **(b)** Anti‐SARS‐CoV‐2 Spike IgM (S‐IgM), IgA (S‐IgA) and IgG (S‐IgG) titres measured as area under the curve (AUC; *n* = 31). **(c)** SARS‐CoV‐2 Spike‐specific, IFN‐γ‐secreting peripheral blood mononuclear cells (S‐IFN‐γ) presented as IFN‐γ spot‐forming units per 10^6^ cells (*n* = 31). **(d)** Spearman's correlation analysis of vaccine response parameters pre‐ and post‐third vaccine dose. **(e, f)** Serological neutralisation of live SARS‐CoV‐2 virus ancestral A.2.2 **(d)** and omicron BA.5 **(e)** strains, pre‐ and post‐booster vaccination. Red dashed lines indicate the target threshold for effective neutralisation correlating with 50% protection from infection. Percentage effective neutralisation is indicated (*n* = 31). **(g)** Overview of changes in sero‐status with a third vaccine dose. Participants are classified by maximum response achieved, where BA.5 LVN > A.2.2 LVN > RBD‐IgG positive > RBD‐IgG negative. **(h–j)** Comparison of humoral immunity following a third vaccine dose in pwMS receiving ocrelizumab (*n* = 31) versus natalizumab (*n* = 10). Treatment groups are compared by percentage of participants achieving seroconversion of anti‐RBD IgG **(h)**, and effective neutralisation of ancestral A.2.2 **(i)** and omicron BA.5 **(j)** variants. **(k)** Relationship between anti‐Spike IgG post‐third vaccination versus days between last ocrelizumab infusion and third vaccine dose administration with Spearman's correlation analysis (*n* = 31). Statistical significance by Wilcoxon‐matched pairs signed rank test (straight bars) or two‐sided Fisher's exact test (crooked bars). Participants with prior SARS‐CoV‐2 infection are represented on graphs but were not included in the statistics presented, nor in statistical significance calculations. ****P < 0.0001; ***P < 0.001; **P < 0.01. A.2.2/BA.5‐LVN, SARS‐CoV‐2 ancestral/omicron BA.5 live virus neutralisation; ns, non‐significant; RBD‐Ig, anti‐SARS‐CoV‐2 receptor‐binding domain immunoglobulin; S‐IFN‐γ, SARS‐CoV‐2 Spike protein‐specific IFN‐γ ELISpot; S‐IgM/A/G, anti‐SARS‐CoV‐2 Spike protein immunoglobulin.

Seroconversion was assessed with the ‘Elecsys Anti‐SARS‐CoV‐2 S’ assay (Roche, Basel, Switzerland). Anti‐receptor‐binding domain (RBD‐) Ig titres pre‐ and post‐third vaccine dose were available for 31 pwMS on ocrelizumab without prior SARS‐CoV‐2 infection. Seroconversion rates (in infection‐naïve participants) increased from 32% to 52% (Figure 1a). A concordant increase was observed in the median titre of anti‐Spike (S‐) IgG (in‐house ELISA; AUC 7.149 vs 160.5, *P* = 0.0096), but not S‐IgM nor S‐IgA (Figure 1b). The third vaccine dose improved T‐cell responses in 24/31 individuals (77%), with the median frequency increasing from 1046 to 1903 spot‐forming units (SFU) per 10^6^ peripheral blood mononuclear cells (*P* < 0.0001, Figure 1c). IFN‐γ T‐cell response did not significantly correlate with measures of antibody response (Figure 1d).

Anti‐nucleocapsid (NC‐) Ig, which is routinely used for assessment of past infection, was below detection in all pwMS on ocrelizumab, including in four individuals with previous PCR positive SARS‐CoV‐2 infections (Supplementary figure 1). The five participants in the natalizumab group with documented prior infection all returned positive NC‐Ig titres (Supplementary figure 1).

### Neutralising antibody responses are inadequate in pwMS receiving ocrelizumab

The capacity of serum to neutralise live SARS‐CoV‐2 virus correlates closely with real‐world protection from infection and disease.^16^ To estimate vaccine effectiveness, Khoury *et al*. defined the relationship between live virus neutralisation titre and risk of infection with SARS‐CoV‐2, and derived a value that correlates with 50% protection from infection [20.2% of the mean neutralising titre of first‐wave convalescent individuals; 95% confidence interval (95% CI) 14.4–28.4%]. To establish an equivalent threshold in the present study, serum samples from 20 first‐wave convalescent individuals were analysed side‐by‐side with the study samples (Supplementary figure 2), and a target threshold for effective neutralisation of IC_50_ = 20 defined (see the *Methods section*). The proportion of pwMS on ocrelizumab achieving this threshold for effective neutralisation of ancestral virus increased from 9.7% to 26% with a third vaccine dose (Figure 1e). Fewer patients demonstrated effective neutralisation of the omicron BA.5 variant, increasing from 0% to 13% with the third dose (Figure 1f). Of those who were seronegative prior to vaccination, 6/21 (29%) seroconverted and 2/21 (9.5%) achieved effective neutralisation (Figure 1g).

Antibody response following the third dose was compared to participants treated with the non‐depleting monoclonal antibody natalizumab. Relative to the natalizumab cohort, pwMS receiving ocrelizumab demonstrated reduced seroconversion of RBD‐Ig (52% vs 100%, *P* = 0.0067; Figure 1h), and significantly lower rates of effective neutralisation of SARS‐CoV‐2 ancestral (26% vs 100%, *P* < 0.0001; Figure 1i) and omicron BA.5 (13% vs 70%, *P* = 0.0004; Figure 1j) variants, despite a longer median time from vaccination to sample collection for the natalizumab group (median: 28 vs 166 days).

During the follow‐up period, 7/34 infection‐naïve participants (21%) from the ocrelizumab group and 2/10 infection‐naïve participants (20%) from the natalizumab group reported contracting COVID‐19. No cases resulted in hospitalisation. All patients in the ocrelizumab group were managed with antivirals. Neither case in the natalizumab group required antivirals.

### Time since ocrelizumab infusion is not associated with vaccine response

Clinical and demographic parameters were assessed for association with antibody responses to the third vaccine dose in the ocrelizumab group. In a multivariate linear regression model, only male sex was associated with antibody response (anti‐RBD Ig: *β* = 131.2, 95% CI 33.99–228.3, *P* = 0.0104; A.2.2 neutralisation: *β* = 73.93, 95% CI 2.745–145.1, *P* = 0.0425; anti‐Spike IgG: *β* = 2843, 95% CI 953–4732, *P* = 0.0056; Supplementary table 1). Notably, time between ocrelizumab infusion and vaccination did not predict, nor correlate with, S‐IgG response to vaccination (*r*_*s*_ = 0.1523, *P* = 0.2277; Figure 1k).

### Immune phenotype associated with ocrelizumab versus natalizumab treatment

**Figure 2:**
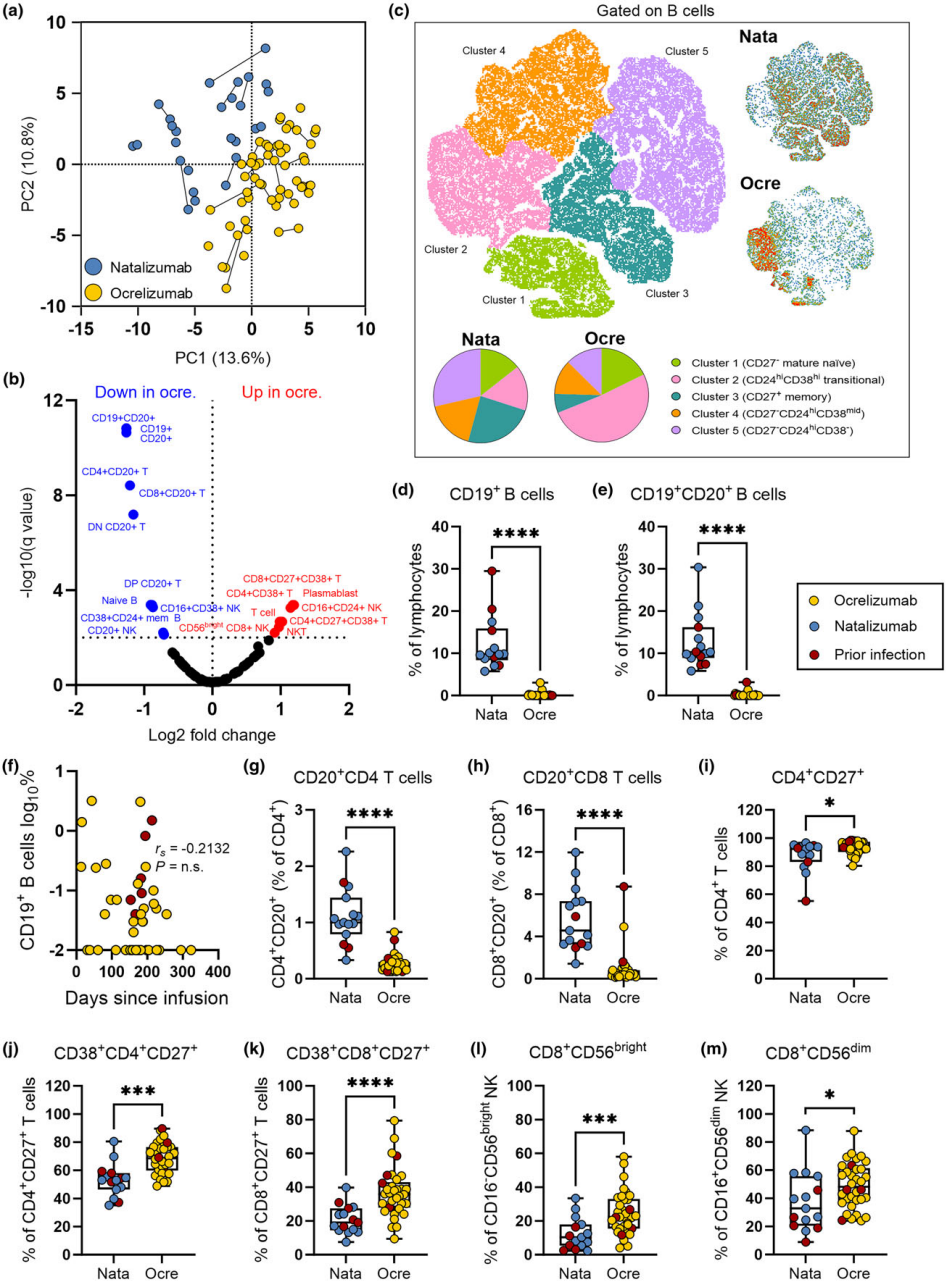
Immune phenotype associated with B‐cell depletion therapy. **(a)** Principal component analysis (PCA) of 101 peripheral blood immune phenotype parameters analysed in people with multiple sclerosis (pwMS) on ocrelizumab pre‐ (*n* = 33) and post‐ (*n* = 24) vaccination or natalizumab pre‐ (*n* = 14) and post‐ (*n* = 13) vaccination. PC1 and PC2 explain 13.6% and 10.8% of variation in immune phenotype, respectively. Paired pre‐ and post‐vaccination samples are joined with a line. **(b)** Volcano plot identifying significant differences (false discovery rate < 1%) in immune phenotype parameters between the ocrelizumab and natalizumab treatment groups. **(c)** Visualisation of B‐cell subset bias in pwMS receiving ocrelizumab versus natalizumab. The t‐distributed stochastic neighbour embedding (tSNE) dimensionality reduction algorithm was applied to concatenated phenotype data for a representative cohort (equal number of ocrelizumab and natalizumab samples and CD19^+^ events), and broad B‐cell subpopulations defined and coloured by FlowSOM clustering. Relative frequencies of B‐cell subpopulations in representative samples from ocrelizumab and natalizumab cohorts are visualised by density plot of equal event number and quantified in pie charts. **(d, e)** Comparison of pre‐vaccination CD19^+^ **(d)** and CD19^+^CD20^+^ **(e)** B‐cell frequencies as a percentage of lymphocytes between ocrelizumab (*n* = 33) and natalizumab (*n* = 14). **(f)** Relationship of days since last ocrelizumab infusion and CD19^+^ B cells (% of lymphocytes) with Spearman's correlation analysis (*n* = 33). **(g–m)** Comparison of selected immune phenotype parameters between ocrelizumab (*n* = 33) and natalizumab (*n* = 14) groups immediately prior to vaccination. T cells were defined as CD3^+^CD19^−^CD14^−^CD56^−^ cells, and natural killer (NK) cells as CD3^−^CD19^−^CD14^−^CD56^+^/CD16^+^ cells, within the physical lymphocyte gate. Differences between treatment groups identified by Mann–Whitney with two‐stage step‐up method to correct for multiple comparisons. *P*‐values for presented comparisons **(d, e, g–l)** are unadjusted: ****P < 0.0001; ***P < 0.001; *P < 0.05. Participants with prior SARS‐CoV‐2 infection (red) were not included in statistical significance calculations.

The severe impairment in protective immunity observed in the ocrelizumab group, and the lack of a relationship between modifiable clinical parameters and vaccine response, prompted us to evaluate the effect of ocrelizumab on immune phenotype to identify immune determinants of vaccine response. One‐hundred and one immune phenotype parameters were assessed in the peripheral blood of pwMS receiving ocrelizumab and natalizumab (Supplementary figures 3–5). Principle component analysis based on these phenotype variables resulted in clustering of patients by treatment, supporting the notion that treatment is the major determinant of immune variation within the cohort (Figure 2a). Eight immune parameters were increased, and 14 decreased, in the ocrelizumab group compared with the natalizumab group, based on a false discovery rate of 1% for significance (Figure 2b). As expected, pwMS on ocrelizumab demonstrated reductions in CD19^+^, CD20^+^ and CD19^+^CD20^+^ B cells as a proportion of lymphocytes (Figure 2b–e). However, B‐cell frequency did not correlate with time since ocrelizumab infusion (Figure 2f).

To visualise differences in the phenotype of B cells between treatment groups, a two‐dimensional plot of concatenated immune phenotype data was constructed using the t‐distributed stochastic neighbour embedding (tSNE) dimensionality reduction algorithm (Figure 2c). B‐cell subpopulation clusters were defined by FlowSOM, and relative frequencies of B‐cell populations were visualised for each treatment group (Figure 2c). The B‐cell compartment in the ocrelizumab cohort was enriched for immature transitional B cells, reflecting a stepwise reconstitution of the B‐cell compartment following depletion (Figure 2c).

In addition to ablation of B cells, proportions of CD20‐expressing populations within the CD4^+^, CD8^+^, double‐positive (CD4^+^CD8^+^) and double‐negative (CD4^−^CD8^−^) T‐cell compartments were significantly reduced (Figure 2b, g and h). CD20^+^CD4^+^ and CD20^+^CD8^+^ T cells, which have been implicated in the pathogenesis of MS,^14^, ^17^, ^18^ represented the greatest difference between groups, with a 4.2‐fold reduction in the proportion of CD4^+^ T cells (geometric mean: 1.0% vs 0.24%, *P* < 0.0001) and a 9.7‐fold reduction in the proportion of CD8^+^ T cells (4.9% vs 0.50%, *P* < 0.0001) expressing CD20 in the ocrelizumab group (Figure 2g and h).

Conversely, the proportion of CD4^+^ T cells expressing the co‐stimulatory receptor CD27, recently proposed as a mechanism underlying the efficacy of B‐cell depletion therapy in MS,^15^ was elevated in the ocrelizumab group (87% vs 93%, *P* = 0.0209; Figure 2i). Expression of the activation marker CD38 was increased in both CD4^+^CD27^+^ (52% vs 68%, *P* < 0.0001) and CD8^+^CD27^+^ (19% vs 34%, *P* < 0.0001) T cells in the ocrelizumab group (Figure 2j and k). These immune populations were stable across pre‐ and post‐vaccination time points in both the ocrelizumab and natalizumab groups, suggesting that differences are associated with treatment and not variations in the timing of sample collection between groups (Supplementary figure 6).

Finally, CD8^+^ NK cells were recently identified as an immunomodulatory cell type associated with reduced risk of relapse in pwMS not receiving treatment.^19^ CD8^+^ NK cells were elevated in the ocrelizumab group as a percentage of both CD56^bright^CD16^−^ immunomodulatory (9.2% vs 21.3%, *P* < 0.0001) and CD56^dim^CD16^+^ conventional NK cells (32% vs 46%, *P =* 0.0284; Figure 2l and m).

### B cells and CD56^bright^ NK cells are associated with vaccine response in pwMS receiving ocrelizumab

**Figure 3:**
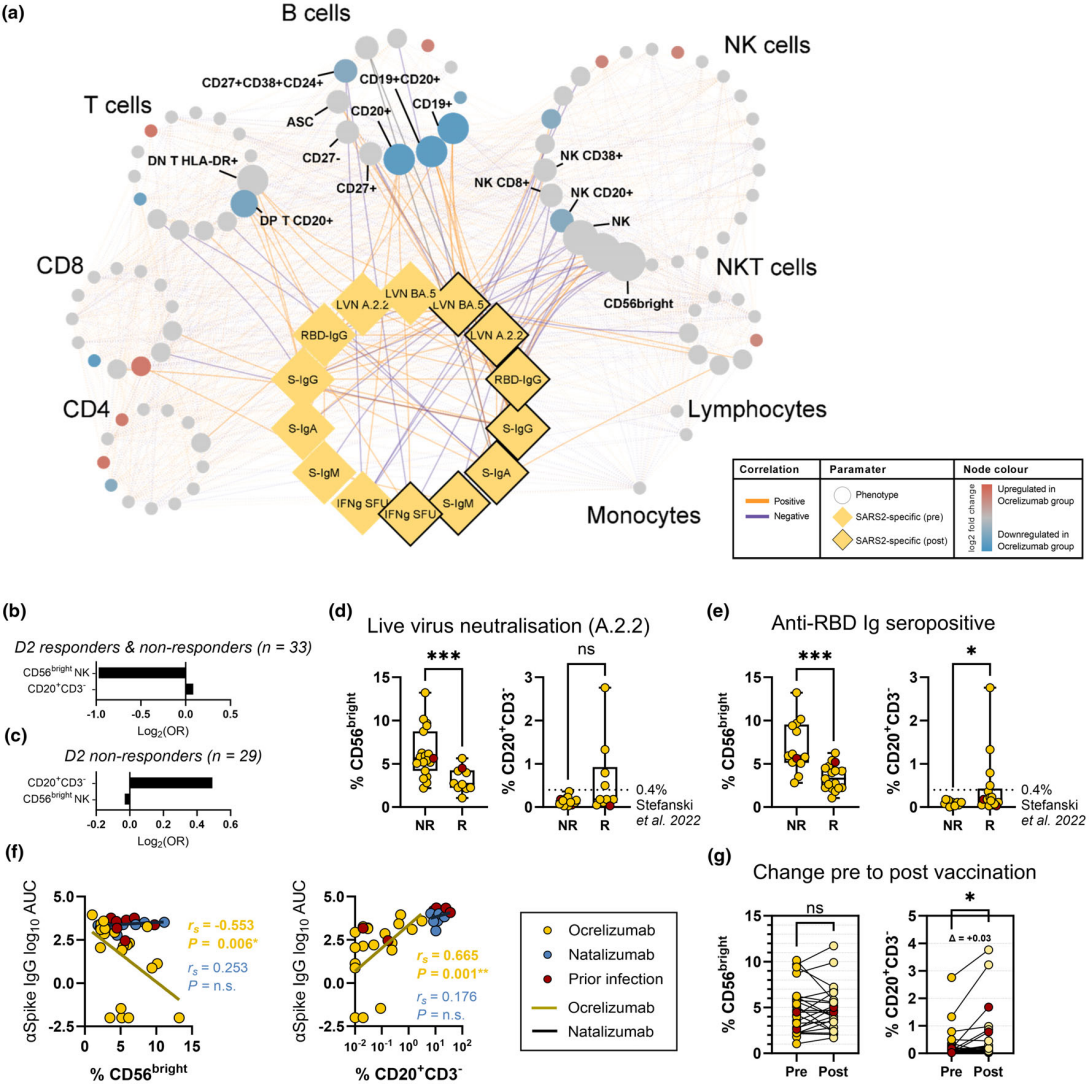
Association of immune phenotype with vaccine response in people with multiple sclerosis (pwMS) receiving ocrelizumab. **(a)** Spearman's correlation network describing the relationship between pre‐vaccination immune phenotype and vaccine response parameters for pwMS receiving ocrelizumab (*n* = 33). Nodes are manually clustered into related immune cell lineages. Node colour denotes differences from the natalizumab group (from Figure 2b). Node size corresponds to the number of edges formed with vaccine response measures. Positive correlations are shown in orange and negative correlations are shown in purple. Phenotype‐to‐response edges are bold, while phenotype‐to‐phenotype edges are faded. Vaccine response measures (yellow diamond nodes) are shown pre‐third dose (no outline) and post‐third dose (black outline). **(b, c)** Pre‐vaccination CD56^bright^ natural killer (NK) cells (% of NK cells) and CD20^+^CD3^−^ cells (% of lymphocytes) were selected from 101 immune phenotype parameters in a penalised LASSO logistic regression model for prediction of effective serological neutralisation of ancestral SARS‐CoV‐2 for the full ocrelizumab cohort (*n* = 33; **b**) and independently for pre‐vaccination non‐responders (*n* = 29; **c**). **(d, e)** Frequencies of CD56^bright^ NK cells (% of NK cells) and CD20^+^CD3^−^ cells (% of lymphocytes) compared between pwMS on ocrelizumab who did and did not achieve effective live virus neutralisation **(d)** or seroconvert anti‐receptor‐binding domain Ig **(e)** following a third vaccine dose. Reconstitution of B cells to 0.4% of lymphocytes has been suggested^20^ as a threshold for seroconversion to SARS‐CoV‐2 vaccination. **(f)** Spearman's correlation of CD56^bright^ NK cells (% of NK cells) and CD20^+^CD3^−^ cells (% of lymphocytes) with anti‐Spike IgG titre (AUC). **(g)** Change in frequency of selected immune cell populations pre‐ to post‐third vaccination (*n* = 24). The change in median CD20^+^CD3^−^ percentage (Δ) is annotated. Statistical significance between groups by Mann–Whitney or between time points by Wilcoxon‐matched pairs signed rank test. ***P < 0.001; *P < 0.05; ns, non‐significant. Participants with prior SARS‐CoV‐2 infection (red) were not included in statistical significance calculations.

As time since ocrelizumab infusion did not predict vaccine response, we assessed whether peripheral immune populations were associated with vaccine response measures. Correlation analysis was performed to describe the relationship between immune phenotype parameters and vaccine response in pwMS receiving ocrelizumab (Figure 3a). Strong positive correlations were observed between B‐cell populations and post‐vaccination humoral immune responses in the ocrelizumab cohort (Figure 3a).

In a penalised multivariate logistic regression model, CD3^−^CD20^+^ B cells (% of lymphocytes) and, unexpectedly, CD56^bright^ NK cells (% of total NK cells) were selected as predictors of effective neutralisation of ancestral SARS‐CoV‐2 by pwMS receiving ocrelizumab with a mean cross‐validation area under the receiver operator characteristic (AUROC) curve of 0.88 (CD56^bright^ NK cells: OR per +1 SD = 0.51; CD3^−^CD20^+^ B cells: OR per +1 SD = 1.06; Figure 3b; Supplementary table 2). The same parameters were selected when regularisation was performed for only the individuals who were non‐responders (A.2.2 LVN < 20) prior to the third dose (mean cross‐validation AUROC = 0.88; CD56^bright^ NK cells: OR per +1 SD = 0.98; CD3^−^CD20^+^ B cells: OR per +1 SD = 1.41; Figure 3c; Supplementary table 3).

CD16^−^CD56^bright^ NK cells at the time of vaccination (both as a proportion of NK cells and as a proportion of lymphocytes) were elevated in individuals who failed to neutralise ancestral virus or seroconvert (Figure 3d and e; Supplementary figure 7A,B), and strongly negatively correlated with anti‐Spike IgG titre (Figure 3f, Supplementary figure 7C). Conversely, participants with pre‐vaccination CD3^−^CD20^+^ B‐cell frequencies ≥ 0.4% of lymphocytes achieved effective neutralisation of ancestral virus (Figure 3d), in line with a previously proposed threshold for B‐cell reconstitution prior to vaccination.^20^ B‐cell (CD20^+^CD19^+^) frequency at the time of vaccination was significantly greater in those who seroconverted (Figure 3e) and correlated strongly with anti‐Spike IgG titre post‐vaccination (Figure 3f). CD3^−^CD20^+^ B‐cell frequency increased over time, consistent with gradual reconstitution of the B‐cell compartment, while CD56^bright^ NK cell frequency was stable across the pre‐ to post‐vaccination time points (Figure 3g). Neither population significantly correlated with anti‐Spike IgG titre in the natalizumab group (Figure 3f).

## Discussion

People receiving BCDT are among the most vulnerable to vaccine‐preventable infections. We sought to identify biomarkers and modifiable determinants of protective immunity to inform future vaccination strategies. Consistent with previous studies,^9^ approximately half of participants were seropositive for SARS‐CoV‐2 RBD‐Ig following a third vaccine dose. The quality of the antibody response in seropositive individuals was largely inadequate; assessed against target viral neutralisation values for protection from infection, 26% of the ocrelizumab cohort demonstrated effective neutralisation of ancestral SARS‐CoV‐2, and 13% against the immune evasive omicron BA.5 variant. Thus, pwMS on BCDT were inadequately protected from COVID‐19 by three vaccine doses.

Booster vaccination improved the median IgG titre and the frequency of functional virus‐specific T cells; however, less than one‐third of seronegative participants seroconverted with a third vaccine dose, and only one participant who was seronegative prior to the third dose achieved effective viral neutralisation. Repeated vaccinations may therefore be beneficial but still inadequate for many people receiving BCDT. Fourth vaccine doses in patient groups receiving BCDT have been reported to result in a further 24–38% of seronegative patients seroconverting.^21^, ^22^ It is worth noting that these studies and others have relied on detection of serum NC‐Ig to identify participants with prior SARS‐CoV‐2 infection. This criterion is flawed as the four participants in our ocrelizumab cohort with PCR‐confirmed SARS‐CoV‐2 did not have detectable NC‐Ig.

Time since infusion has been suggested as a key modifiable determinant of vaccine response, with vaccine administration at the end of an infusion cycle recommended. The standard dosing interval for ocrelizumab is 6 months, and some centres implemented delayed infusions to maximise B‐cell reconstitution prior to vaccination. A relationship between time since ocrelizumab infusion and antibody response has been reported previously.^23^, ^24^ However, these associations were driven by individuals who were more than 6 months post‐infusion at the time of vaccination, while B‐cell reconstitution (≥ 1 cell/μL) and seroconversion were rare in individuals *within* 6 months of infusion.^24^ Our findings suggest that varying timing of vaccination within the standard 6 month infusion interval is unlikely to affect antibody responses.

The problem of inadequate vaccine response in this cohort extends beyond COVID‐19, and raises the potential for personalised approaches to vaccination. Several studies have suggested B‐cell reconstitution as a biomarker of response, with 40 B cells/μL^11^ (approximately 1.6% of lymphocytes) suggested as a pre‐vaccination target in pwMS, and 10 B cells/μL (approximately 0.4% of lymphocytes) suggested in a study of rheumatoid arthritis patients receiving B‐cell depletion,^20^ although three quarters of participants were > 6 months post‐infusion. The latter value is supported by the present study in which all four participants with CD3^−^CD20^+^ B‐cell frequencies > 0.4% of lymphocytes achieved seroconversion and neutralisation titres estimated to confer 50% protection from infection against ancestral SARS‐CoV‐2 virus.

The majority of responders in our study were able to achieve effective viral neutralisation despite having low pre‐vaccination B‐cell frequencies. Comprehensive evaluation of multiple immune phenotypes identified a novel predictor of antibody response in CD56^bright^ NK cells, which inversely correlated with antibody titre and neutralisation and, in conjunction with B‐cell reconstitution in a multiple logistic regression model, considerably improved stratification of responders. CD56^bright^ NK cells are an immunomodulatory population of NK cells, which are cytotoxic towards proliferating and autoreactive CD4^+^ T cells *in vitro*.^25^, ^26^, ^27^, ^28^, ^29^ In MS, they have been reported to be elevated in the cerebrospinal fluid (CSF) and present in periventricular lesions of the brain, although their role is unclear.^30^, ^31^ Several DMTs (not including ocrelizumab and natalizumab) have been reported to alter the frequency of CD56^bright^ NK cells in the periphery,^32^, ^33^, ^34^, ^35^, ^36^, ^37^, ^38^, ^39^, ^40^, ^41^, ^42^, ^43^, ^44^, ^45^ and an increase (or lack of decrease) in CD56^bright^ NK cells has been associated with response to treatment with IFNβ, dimethyl fumarate, fingolimod and daclizumab,^34^, ^35^, ^46^, ^47^, ^48^ suggesting a potential immunoregulatory role in MS. In this study, CD56^bright^ NK cells were negatively associated with antibody response to vaccination in the ocrelizumab treatment group but not the natalizumab group. This may be due to the smaller sample size in the natalizumab group. Alternatively, a negative influence of CD56^bright^ NK cells on the antibody response may be imperceptible under normal circumstances and is unmasked when B‐cell frequencies are low.

Our study had limitations. As a result of a difference in the time from vaccination to sample collection between treatment groups, the precise effect of B‐cell depletion on antibody titres and T‐cell responses was not assessed. Additionally, absolute cell counts were not measured. While all immune subpopulations were quantified as a proportion of the parent population, lineages were quantified as a proportion of lymphocytes and were thus influenced by B‐cell frequencies in the ocrelizumab group. The conclusions of the study are also limited by the lack of a validation cohort. Prospective studies will be required to validate and determine accurate effect sizes for B‐cell and CD56^bright^ NK cells as biomarkers of antibody response to vaccination, as well as reproducibility in other populations and vaccines. If validated, further studies will be required to shed light on the mechanistic relationship between CD56^bright^ NK cells, antibody production and MS disease pathogenesis.

Collectively, we highlight severe immune impairment in pwMS on BCDT and provide insights into the changes in immune phenotype associated with ocrelizumab use relevant to disease pathogenesis and how the immune phenotype relates to aspects of cellular and humoral response to vaccination. The data presented here support the use of prophylactic measures for protection against COVID‐19 in pwMS receiving BCDTs and support the potential of immune phenotype markers for the personalisation of vaccination strategies in this highly vulnerable group.

## Methods

### Standard protocol approvals, registrations and participant consents

The study was approved by the Central Adelaide Local Health Network Human Research Ethics Committee (approval number: 15083), and conducted from November 2021 to August 2022 (ACTRN12623001249640). Study participants were recruited from the Royal Adelaide Hospital Specialty Vaccination Clinic and informed consent was provided by all participants. Inclusion criteria were the ability to understand requirements of the study, to provide written informed consent and to attend follow‐up; age 18+ years; diagnosis of RRMS, PPMS and SPMS; receiving ocrelizumab or natalizumab as primary treatment with the last dose received within 24 months of vaccine administration; have elected to receive a third COVID‐19 vaccine dose with a Therapeutic Goods Administration (TGA) approved mRNA‐platform vaccine. Past SARS‐CoV‐2 infection was determined by record of positive nasopharyngeal swab by PCR and confirmed via verbal history taken from all participants. All participants were followed up by telephone February 2023 (approximately 6 months post‐final study visit), and incidence and severity of COVID‐19 (hospitalisation and treatment required) was self‐reported at this time and confirmed with electronic medical records.

### SARS‐CoV‐2 spike protein production and ELISA

Anti‐SARS‐CoV‐2 Spike IgM, IgA and IgG were quantified by in‐house ELISA, as previously described.^49^, ^50^ Plates were coated with prefusion SARS‐CoV‐2 Spike ectodomain (isolate WHU1, residues 1‐1208) with HexaPro mutations (kindly provided by Dr Adam Wheatley),^49^ and participant sera serially diluted, and end point titres calculated and expressed as area under the curve (AUC). AUC calculations were performed using Prism v9.0.0 (GraphPad Software Inc.).

Anti‐SARS‐CoV‐2 Spike RBD and NC Ig titres were quantified using the ‘Elecsys Anti‐SARS‐CoV‐2 S’ and ‘Elecsys Anti‐SARS‐CoV‐2’ assays on the Cobas system (Roche), through the state pathology service, SA Pathology. The quantitation range for detection of anti‐RBD Ig in this assay is 0.4–250 U/mL. This assay is widely available through pathology services globally and can be compared with other assays by conversion to WHO Standard International Units, where 1 U/mL is comparable to ~1.029 BAU/mL.^51^

### SARS‐CoV‐2 live virus neutralisation assay

Rapid high‐content neutralisation assay with HEK‐ACE2/TMPRSS cells, HAT‐24 cells was performed as previously described.^52^, ^53^ HAT‐24 cells were seeded in 384‐well plates at 16 × 10^3^ cells/well in the presence of the live cell nuclear stain Hoechst‐33342 dye (NucBlue; Invitrogen) at a concentration of 5% v/v. Twofold dilutions of patient serum samples were mixed with an equal volume of SARS‐CoV‐2 virus solution standardised at 2xVE_50_ and incubated at 37°C for 1 h before adding 40 μL, in duplicate, to the cells. Viral variants used were the omicron sub‐variant BA.5, and the ‘ancestral’ virus (A.2.2) from clade A and presenting no amino acid mutations in Spike (similar to Wuhan ancestral variant and vaccine strain). Plates were incubated for 24 h post‐infection and entire wells were imaged by high‐content fluorescence microscopy, cell counts obtained with automated image analysis software, and the percentage of virus neutralisation was calculated with the formula: %N = (D−(1−Q)) × 100/D, as previously described.^52^, ^53^ Sigmoidal dose–response curves and IC_50_ values (reciprocal dilution at which 50% neutralisation is achieved) were calculated with Prism v9.0.0 (GraphPad Software Inc.).

### Defining a target threshold for effective neutralisation

The threshold for effective neutralisation was derived from a published value of 20.2% of the mean neutralisation titre (IC_50_) of first‐wave convalescent individuals.^16^ This value correlated with 50% protection from real‐world infection early in the COVID‐19 pandemic. In our study, 20 convalescent sera individuals infected with SARS‐CoV‐2 Wuhan between 1 March 2020 and 30 April 2020 in South Australia (CALHN HREC approval number: 13050)^49^ were included in the same experimental run as the study samples, and 20.2% of the mean IC_50_ for this group calculated to be 14.7 (95% CI 10.5–20.7). As the lowest dilution factor tested for all samples was 20, and this was within the 95% CI calculated by Khoury *et al*., effective neutralisation of both the ancestral and omicron BA.5 variants was defined as an IC_50_ ≥ 20. While the present study extrapolates the same value for ‘effective neutralisation’ to the analysis of omicron BA.5, an equivalent correlate for the omicron BA.5 variant has not been published. This endpoint is consistent with previous SARS‐CoV‐2 vaccination trials.^54^, ^55^

### IFN‐γ ELISpot

IFN‐γ ELISpots were performed in‐house, consistent with endpoints for similar trials of SARS‐CoV‐2 vaccine response.^56^, ^57^, ^58^, ^59^ Briefly, multiscreen‐IP HTS plates (Merck Millipore) were coated with anti‐human IFN‐γ (clone 2G1; Thermo Fisher). Peripheral blood mononuclear cells were thawed by dropwise addition of complete media (20% FCS) with Benzonase® nuclease (Merck Millipore) to prevent aggregation and stimulated for 18 h with four pools of overlapping peptides spanning the entire length of the spike glycoprotein of the USA‐WA1/2020 strain. Individual peptides are 17‐ or 13‐mers, with 10 amino acid overlaps, obtained through BEI Resources, NIAID, NIH (*Peptide Array, SARS‐Related Coronavirus 2 Spike (S) Glycoprotein, NR‐52402*). Secreted IFN‐γ was detected with anti‐human IFN‐γ:biotin (Clone B133.5; Thermo Fisher) followed by streptavidin:HRP (BD Biosciences) and AEC substrate (BD Biosciences). Developed spots were counted automatically by use of an ELISpot reader (Cellular Technology Ltd., Bonn, Germany), background (untreated well counts) subtracted from stimulated wells, and counts presented as the sum of the four pools per 10^6^ cells.

### Immune phenotyping

Immune phenotyping was performed using a previously described and validated panel/protocol.^49^ Briefly, thawed PBMCs were stained in a U‐bottom plate with 30 μL antibody master‐mix for 20 min in the dark. Stained PBMCs were washed twice with 200 μL FACS wash, centrifuged at 300 × g for 5 min and fixed with 200 μL FACS Fix (2% paraformaldehyde in PBS) for 20 min at RT in the dark. Fixed cells were then centrifuged at 300 × g for 5 min, washed in 200 μL FACS wash, then spun at 300 × g for 5 min and resuspended in 50 μL FACS wash for analysis using the BD FACS Symphony within 24 h. Details of monoclonal antibodies used are available in Supplementary table 4.

Staining and acquisition was performed in three batches. To control for batch effects, the BD FACS symphony lasers were calibrated with dye conjugated standards (Cytometer Set &Track beads) before each run. All samples were acquired with all 28 PMTs recording events. All PMT voltages were adjusted to unstained negative control baseline, typically log scale 10^2^. Antibodies were titrated for optimal signal‐over‐background so that single positive stains sat within log scale 10^3^–10^5^ of designated PMT. Compensation was set with beads matched to each panel antibody combination using spectral compensation using the FlowJo Software v10 (BD Biosciences). Exported FCS files had compensation values adjusted manually post‐acquisition on a file‐by‐file basis in FCS Express v6 (*De Novo* Software) and gates manually adjusted between batches as required. Once compensated, low data quality events were excluded based upon time acquired (at the sample acquisition start and before sample exhaustion), with further time exclusion gates based on blockages or unexplained loss of events for a period of time during acquisition. Events positive for LIVE/DEAD staining were removed, and events were gated for FSC‐H/‐A as well as SSC‐H/‐A linearity, and restricted FSC‐W and SSC‐W values for doublet discrimination. Live single cells were then broadcast on a SSC‐A/FSC‐A plot to determine size and complexity. Lymphocytes and monocytes were quantified as a percentage of viable single cells; lineages (e.g. CD20+ B cells) were quantified as a percentage of viable lymphocytes (defined by FSC/SSC); and subpopulations (e.g. CD56^bright^ NK cells) were quantified as a percentage of their parent gate, unless otherwise indicated (Supplementary figures 3–5).

### Statistics and data visualisation

All statistical analyses were performed using Prism v9.0.0 (Graphpad Software Inc.), Stata Statistical Software: Release 14.2 (StataCorp) or R v4.3.1. All tests were two‐tailed and no assumptions were made about the distribution of the data sets; non‐parametric tests were used in all cases for comparisons. Accordingly, Mann–Whitney and Kruskal–Wallis tests with Dunn's correction were applied to pair‐wise and multiple comparisons, respectively.

#### Exploratory comparisons

Differences in pre‐vaccination immune phenotype parameters between treatment groups were identified by Multiple Mann–Whitney tests, with two‐stage step‐up method employed to correct for multiple comparisons. Statistical significance was determined as a false discovery rate (FDR) < 0.01.

#### Regression modelling

Multiple linear regression by least squares modelling was applied to post‐vaccination antibody titres (S‐IgM, S‐IgA, S‐IgG and RBD‐Ig), neutralisation data (A.2.2 and omicron BA.5) and ELISpots, as well as change in ELISpot counts pre‐ to post‐vaccination for the ocrelizumab cohort. Assumptions of normality of residuals were tested using D'Agostino‐Pearson omnibus (K2) and of linearity using visual inspection of scatter plots. Variables included were age, sex (M/F), time between infusion and vaccination, primary course vaccine type (BNT162b2/ChAdOx1), and time between second and third dose. Association of pre‐existing immunity (i.e. pre‐vaccination antibody and neutralisation titres and ELISpot counts) with change in ELISpot count pre‐ to post‐vaccination was assessed in a separate multiple linear regression analysis. Multiple linear regression modelling was conducted using Prism v9.0.0 (GraphPad Software Inc.). Association between immune phenotype parameters and vaccine response was assessed by multiple logistic regression for a binary outcome of effective neutralisation of SARS‐CoV‐2 A.2.2 (IC_50_ ≥ 20). Covariate selection was achieved with penalised LASSO regularisation for logistic regression using the Glmnet package (v4.1–7) in R, which reduces coefficients of multi‐collinear and non‐significant variables to zero. This approach resulted in a multiple logistic regression model for CD20^+^ B‐cell and CD56^bright^ NK cell frequencies, with a conservative (penalised) odds ratio estimate. A limitation of the regularisation approach is that a meaningful *P*‐value and CI cannot be estimated.

#### Dimensionality reduction

For visual comparison of B‐cell phenotype between treatment groups, phenotype data (FCS files) for 13 ocrelizumab and 13 natalizumab samples acquired in the same batch were concatenated. CD19^+^ events from natalizumab samples were downsampled such that the analysis included an equal number of B‐cell events from each treatment group. Dimensionality reduction parameters by tSNE were estimated based on 100 000 events (Perplexity: 90; Iterations: 1500) for major B‐cell parameters (CD24, CD38 and CD27) using FCS Express v6 (*De Novo* Software), and five FlowSOM clusters defined using the FlowJo Software v10 (BD Biosciences). Concatenated data from the representative samples were displayed as a coloured dot plot, and the relative frequencies of B‐cell subsets for each treatment group compared by side‐by‐side density plots (of equal event count) and pie charts. As a representative figure, no statistical analysis was applied.

#### Spearman's correlation analysis

Spearman correlations were performed using Prism v9.0.0 (GraphPad Software Inc.). Relationships between pre‐vaccination immune phenotype variables, vaccine response measures, and changes in immune phenotype associated with treatment in the ocrelizumab group were visualised using Cytoscape v3.9.1. Network edges between immune phenotype parameters and vaccine response measures were constructed for significant (*P* < 0.05) Spearman's rank correlation coefficients (*r*_*s*_) of < −0.3 and > 0.3, and immune phenotype nodes sized and coloured to reflect relatedness (number of shared edges/correlations) with vaccine response measures and significant differences to the natalizumab cohort, respectively.

## Author contributions

GBP, JR and PH: Conceptualisation. GBP, MJT, CSC, CMH, ST and BGB: Methodology. GBP, CMH, MJT, SS and AELY: Formal analysis. GBP, CMH, CSC, MJT, AELY, JDZ, AAg, VM and AAk: Investigation. GBP: Visualisation; writing – original draft. GBP, SCB, BGB, PTC and PH: Funding acquisition. KW, JY, MBR and PH: Project administration. PRH, CMH, MGM, PH, SCB, BGB and PTC: Supervision. All authors reviewed the manuscript and contributed to the writing – review and editing.

## Conflict of interest

The authors declare no conflict of interest.

## Funding

This work has received funding from The Hospital Research Foundation Group (outside of structured grant round) and the Royal Adelaide Hospital Research Committee (RRC)/Health Services Charitable Gifts Board (HSCGB; project grant, 70‐05‐52‐05‐20). GBP and MJT received support from the Mary Overton Research Fellowship (RRC/HSCGB) and Jacquot Research Scholarship (Royal Australasian College of Physicians), respectively. BGB and MGM were supported by THRFG Mid‐Career and Early‐Career Fellowships, respectively.

## Supporting information


Supplementary table 1
Supplementary table 2
Supplementary table 3
Supplementary table 4
Supplementary figure 1
Supplementary figure 2
Supplementary figure 3
Supplementary figure 4
Supplementary figure 5
Supplementary figure 6
Supplementary figure 7

## Data Availability

Phenotype and correlation data are available as a network (SIF) file, and raw values can be obtained by reasonable request to the corresponding author.
