# Supplementary material for: CD56bright NK cells are negatively associated with antibody response to vaccination in people with multiple sclerosis on B‐cell‐depleting therapy
Source: Clin Transl Immunology. 2026 Feb 10;15(2):e70079. doi: 10.1002/cti2.70079 (PMC12887676; doi:10.1002/cti2.70079)
Supplement: Supplementary file 1 — Supplementary table 1 Supplementary table 2 Supplementary table 3 Supplementary table 4 Supplementary figure 1 Supplementary figure 2 Supplementary figure 3 Supplementary figure 4 Supplementary figure 5 Supplementary figure 6 Supplementary figure 7 [file CTI2-15-e70079-s001.docx]

**Supplementary Data for Perkins *et al***

**Supplementary Table 1.** Multivariable linear regression of clinical factors associated with antibody response to vaccination in people with multiple sclerosis receiving ocrelizumab. Unstandardised β coefficients (95% CI) are reported. Predictors included: age (years), sex (male vs female), days since last ocrelizumab infusion to dose 3, primary course vaccine (ChAdOx1 vs BNT162b2), and days between dose 2 and dose 3.

**A.2.2 Neutralisation (IC50)**; N = 27; R² = 0.181; Adjusted R² = -0.014

| Predictor | β (95% CI) | p-value |
| --- | --- | --- |
| Sex: male (vs female) | 73.93 (745.0 to 145.10) | **0.0425** |
| Age (per year) | -0.09 (-2.59 to 2.41) | 0.9428 |
| Days since last infusion to dose 3 (per day) | 0.12 (-0.30 to 0.55) | 0.5525 |
| Primary course: ChAdOx1 (vs BNT162b2) | -18.81 (-139.01 to 101.39) | 0.748 |
| Days between dose 2 and 3 (per day) | 0.21 (-0.40 to 0.81) | 0.4798 |

**BA.5 Neutralisation (Endpoint titre)**; N = 27; R² = 0.193; Adjusted R² = 0.001

| Predictor | β (95% CI) | p-value |
| --- | --- | --- |
| Sex: male (vs female) | 11.59 (0.25 to 22.92) | 0.0556 |
| Age (per year) | -0.07 (-0.45 to 0.32) | 0.724 |
| Days since last infusion to dose 3 (per day) | 0.02 (-0.05 to 0.08) | 0.6232 |
| Primary course: ChAdOx1 (vs BNT162b2) | -0.79 (-19.37 to 17.80) | 0.9307 |
| Days between dose 2 and 3 (per day) | 0.01 (-0.08 to 0.10) | 0.8474 |

**ELISpot (spots per 10^6^ PBMCs)**; N = 31; R² = 0.032; Adjusted R² = -0.161

| Predictor | β (95% CI) | p-value |
| --- | --- | --- |
| Sex: male (vs female) | -495.24 (-1995.34 to 1004.86) | 0.5028 |
| Age (per year) | -6.45 (-57.70 to 44.79) | 0.7975 |
| Days since last infusion to dose 3 (per day) | 1.36 (-7.46 to 10.18) | 0.7541 |
| Primary course: ChAdOx1 (vs BNT162b2) | -4.02 (-2647.39 to 2639.35) | 0.9975 |
| Days between dose 2 and 3 (per day) | 2.62 (-9.79 to 15.03) | 0.6675 |

**Anti-RBD Ig (U/mL)**; N = 28; R² = 0.344; Adjusted R² = 0.194

| Predictor | β (95% CI) | p-value |
| --- | --- | --- |
| Sex: male (vs female) | 131.2 (33.99 to 228.30) | **0.0104** |
| Age (per year) | 0.15 (-3.09 to 3.39) | 0.9246 |
| Days since last infusion to dose 3 (per day) | -0.20 (-0.74 to 0.34) | 0.4469 |
| Primary course: ChAdOx1 (vs BNT162b2) | -71.94 (-227.86 to 83.99) | 0.3491 |
| Days between dose 2 and 3 (per day) | 0.53 (-0.26 to 1.31) | 0.176 |

**Anti-Spike IgA (AUC)**; N = 23; R² = 0.257; Adjusted R² = 0.039

| Predictor | β (95% CI) | p-value |
| --- | --- | --- |
| Sex: male (vs female) | 716.17 (-205.34 to 1637.69) | 0.1194 |
| Age (per year) | 5.27 (-28.77 to 39.31) | 0.7479 |
| Days since last infusion to dose 3 (per day) | 1.64 (-3.33 to 6.61) | 0.4957 |
| Primary course: ChAdOx1 (vs BNT162b2) | -334.80 (-1818.22 to 1148.63) | 0.64 |
| Days between dose 2 and 3 (per day) | 7.84 (-2.59 to 18.27) | 0.1311 |

**Anti-Spike IgG (AUC)**; Model fit: N = 23; R² = 0.415; Adjusted R² = 0.243

| Predictor | β (95% CI) | p-value |
| --- | --- | --- |
| Sex: male (vs female) | 2842.57 (952.85 to 4732.29) | **0.0056** |
| Age (per year) | 16.05 (-53.75 to 85.85) | 0.6338 |
| Days since last infusion to dose 3 (per day) | 4.76 (-5.42 to 14.95) | 0.3376 |
| Primary course: ChAdOx1 (vs BNT162b2) | -493.95 (-3535.97 to 2548.07) | 0.7361 |
| Days between dose 2 and 3 (per day) | 6.75 (-14.63 to 28.14) | 0.5143 |

**Anti-Spike IgM (AUC)**; N = 23; R² = 0.195; Adjusted R² = -0.041

| Predictor | β (95% CI) | p-value |
| --- | --- | --- |
| Sex: male (vs female) | 1.47 (-0.46 to 3.41) | 0.127 |
| Age (per year) | -0.02 (-0.09 to 0.06) | 0.6604 |
| Days since last infusion to dose 3 (per day) | -0.00 (-0.01 to 0.01) | 0.9625 |
| Primary course: ChAdOx1 (vs BNT162b2) | -0.45 (-3.57 to 2.66) | 0.7628 |
| Days between dose 2 and 3 (per day) | 0.01 (-0.01 to 0.03) | 0.4632 |

**Supplementary Table 2.** Association of pre-vaccination immune phenotype with effective neutralisation response (SARS-CoV-2 A.2.2) to a third vaccine dose in people with multiple sclerosis receiving ocrelizumab. L1-penalised (LASSO) logistic regression was used to model responder (R) vs non-responder (NR) status. Predictors were standardised (z-score) prior to model fitting. Penalty strength was selected by 5-fold stratified cross-validation to maximise AUROC. The stability of selected features (non-zero coefficients: CD56^bright^ NK cells as a percentage of NK cells, CD20^+^CD3^-^ B cells as a percentage of lymphocytes) was evaluated by bootstrapping (1,000 resamples). Odds ratios are reported per +1 standard deviation change in the predictor.

LASSO logistic regression (L1-penalised); N = 33 (R: 11, NR: 22); 5-fold stratified CV; Best C = 0.94; Mean CV AUROC = 0.875; z-score standardised.

| **Selected feature** | **Standardised coef.** | **OR per +1 SD** | **Direction** | **Bootstrap selection frequency** |
| --- | --- | --- | --- | --- |
| CD56^bright^ NK cells | -0.676 | 0.51 | Higher = lower odds of response | 0.80 |
| CD20^+^CD3^-^ B cells | 0.058 | 1.06 | Higher = higher odds of response | 0.29 |

**Supplementary Table 3.** Association of pre-vaccination immune phenotype with effective neutralisation response (SARS-CoV-2 A.2.2) to a third vaccine dose in people with multiple sclerosis receiving ocrelizumab *who lacked effective neutralisation following dose 2*. L1-penalised (LASSO) logistic regression was used to model responder (R) vs non-responder (NR) status. Predictors were standardised (z-score) prior to model fitting. Penalty strength was selected by 5-fold stratified cross-validation to maximise AUROC. The stability of selected features (non-zero coefficients: CD56^bright^ NK cells as a percentage of NK cells, CD20^+^CD3^-^ B cells as a percentage of lymphocytes) was evaluated by bootstrapping (500 resamples). Odds ratios are reported per +1 standard deviation change in the predictor.

LASSO logistic regression (L1-penalised); N = 29 (R: 7, NR: 22); 5-fold stratified CV; Best C = 0.94; Mean CV AUROC = 0.875; z-score standardised.

| **Selected feature** | **Standardised coef.** | **OR per +1 SD** | **Direction** | **Bootstrap selection frequency** |
| --- | --- | --- | --- | --- |
| CD20^+^CD3^-^ B cells | 0.341 | 1.406 | Higher = higher odds of response | 0.696 |
| CD56^bright^ NK cells | -0.021 | 0.979 | Higher = lower odds of response | 0.264 |

**Supplementary Table 4.** Details of monoclonal antibodies used for immune phenotyping of peripheral blood mononuclear cells.

| **Antigen** | **Clone** | **Fluorochrome** | **Company** | **Catalogue number** | **Concentration** |
| --- | --- | --- | --- | --- | --- |
| CD8 | RPA-T8 | BUV395 | BD Biosciences | 563795 | 1:3 |
| CD4 | SK3 | BUV496 | BD Biosciences | 612936 | 1:12 |
| CD3 | UCHT1 | BUV737 | BD Biosciences | 612750 | 1:4 |
| CD38 | HIT2 | BV421 | BD Biosciences | 562444 | 1:5 |
| CD183/CXCR3 | 1C6 | BV650 | BD Biosciences | 740603 | 1:19 |
| CD19 | SJ25C1 | BV711 | BD Biosciences | 563036 | 1:18 |
| CD14 | M5E2 | FITC | BD Biosciences | 555397 | 1:21 |
| CD56/NCAM | HCD56 | PerCP-Cy5.5 | Biolegend | 318322 | 1:20 |
| CD16 | 3G8 | PE | BD Biosciences | 556619 | 1:12 |
| CD24 | ML5 | PE-CF594 | BD Biosciences | 562405 | 1:3 |
| HLA-DR | G46-6 | APC | BD Biosciences | 559866 | 1:18 |
| Live/Dead | Stain | FVS700 | BD Biosciences | 564997 | 1:2 |
| CD20 | 2H7 | APC-H7 | BD Biosciences | 560853 | 1:21 |
| CD27 | M-T271 | PE-Cy7 | BD Biosciences | 560609 | 1:5 |

***
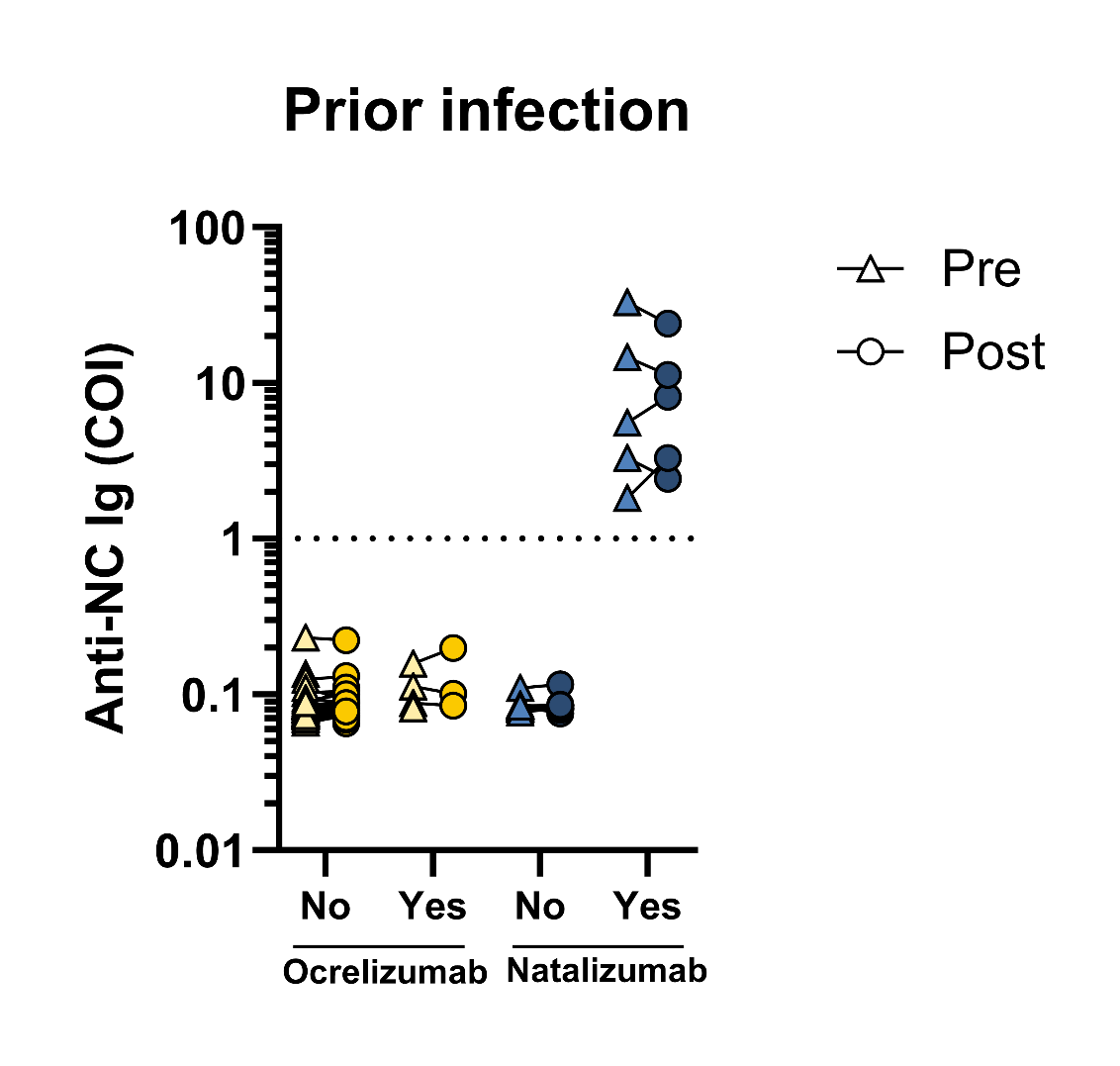
***

**Supplementary Figure 1.** Anti-SARS-CoV-2 nucleocapsid (NC) Ig (Elecsys Anti-SARS-CoV-2, Roche) does not identify people with multiple sclerosis (pwMS) receiving ocrelizumab who had prior SARS-CoV-2 infection. PwMS receiving ocrelizumab who had a PCR-confirmed SARS-CoV-2 infection prior to sample collection (n = 4) remained negative for anti-nucleocapsid Ig (cutoff index < 1.0), while those receiving natalizumab (n =5) returned positive results (cutoff index > 1.0).

**
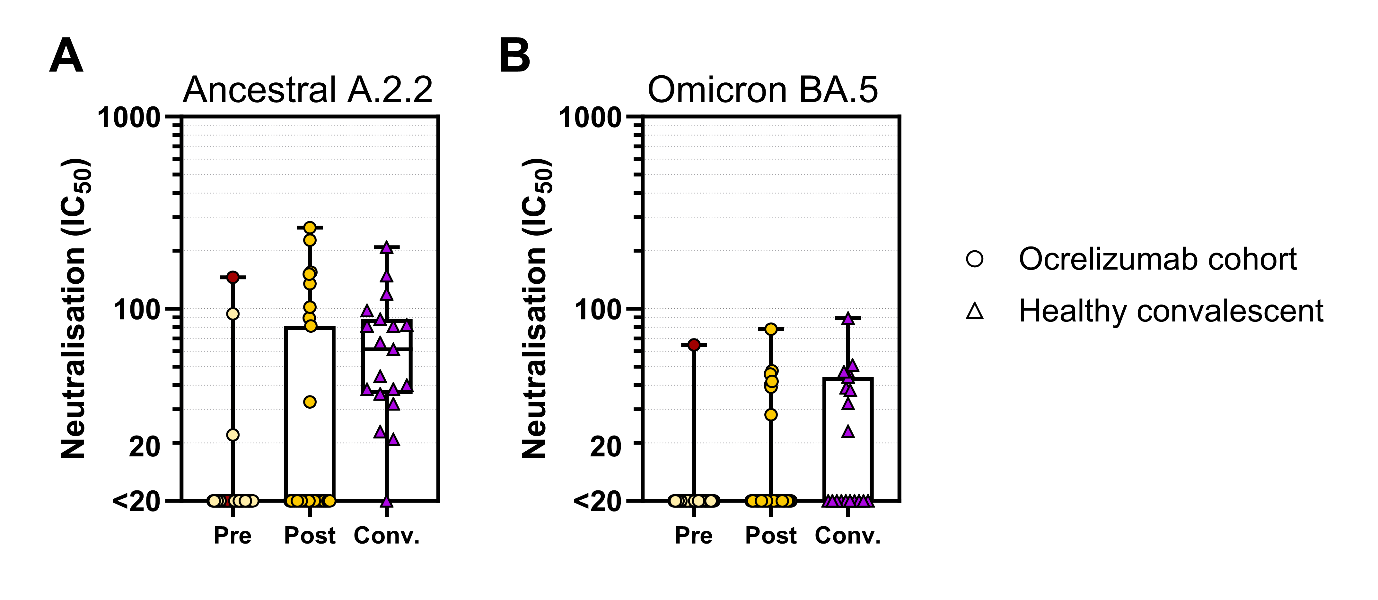

Supplementary Figure 2.** Live virus neutralisation titres for healthy convalescent individuals infected with SARS-CoV-2 Wuhan strain between 1 March 2020 and 30 April 2020. IC50 values were used to define the target threshold for effective neutralisation that was applied in this study. Specifically, a target threshold of IC50 ≥ 20 was defined based on 20.2% of the mean neutralisation titre of the convalescent cohort, in line with the correlate of 50% protection from infection from SARS-CoV-2 Wuhan reported by Khoury *et al.*^16^ Pre and post-vaccination titres for the ocrelizumab cohort (Figure 1E,F) are shown for comparison and individuals who were previously infected with SARS-CoV-2 are shown in red.


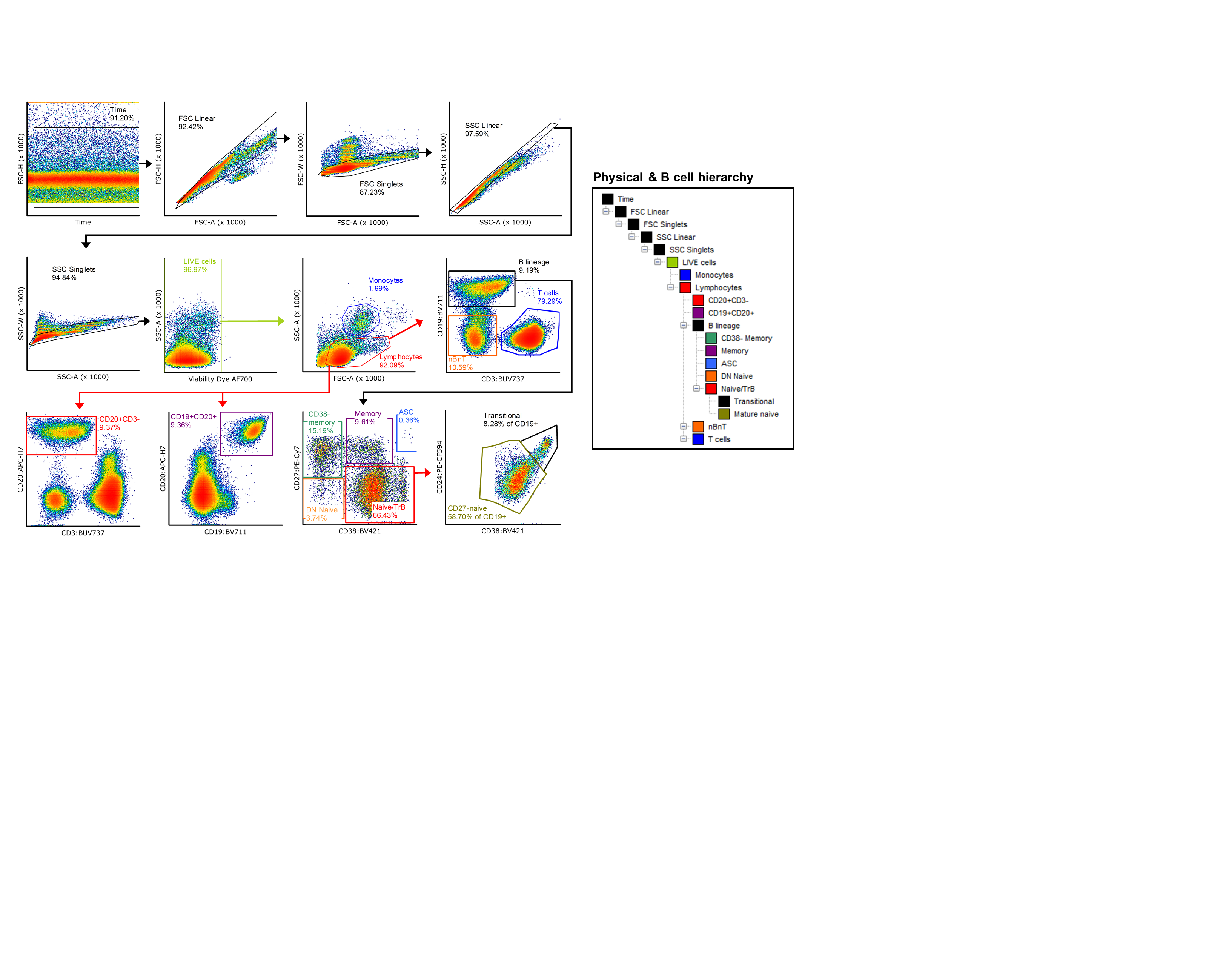


**Supplementary Figure 3.** Flow cytometry gating strategy used to define viable single cells and B cell subpopulations.


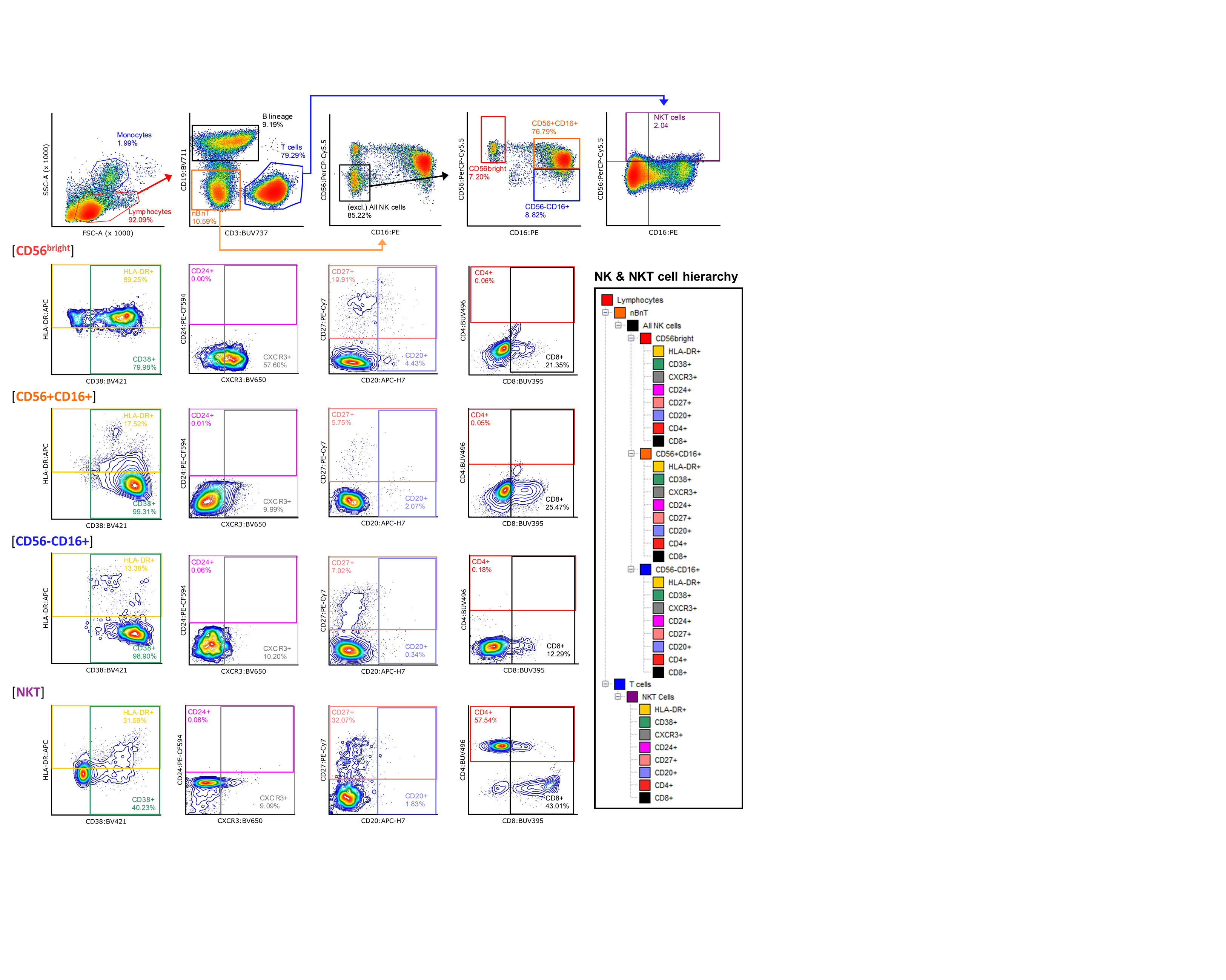


**Supplementary Figure 4.** Flow cytometry gating strategy used to define natural killer cell and natural killer T cell populations.

**Supplementary Figure 5.** Flow cytometry gating strategy used to define T cell populations.


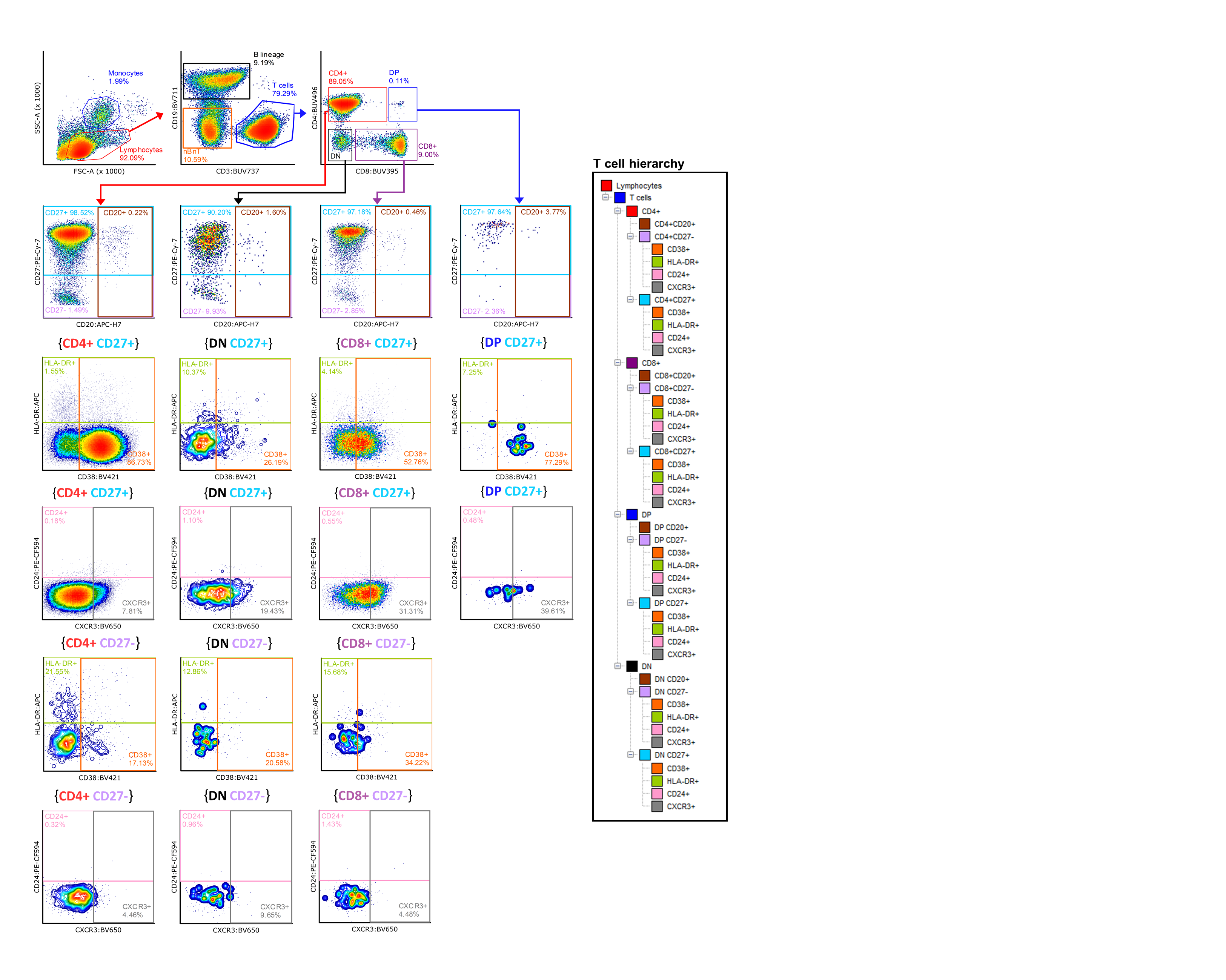


**
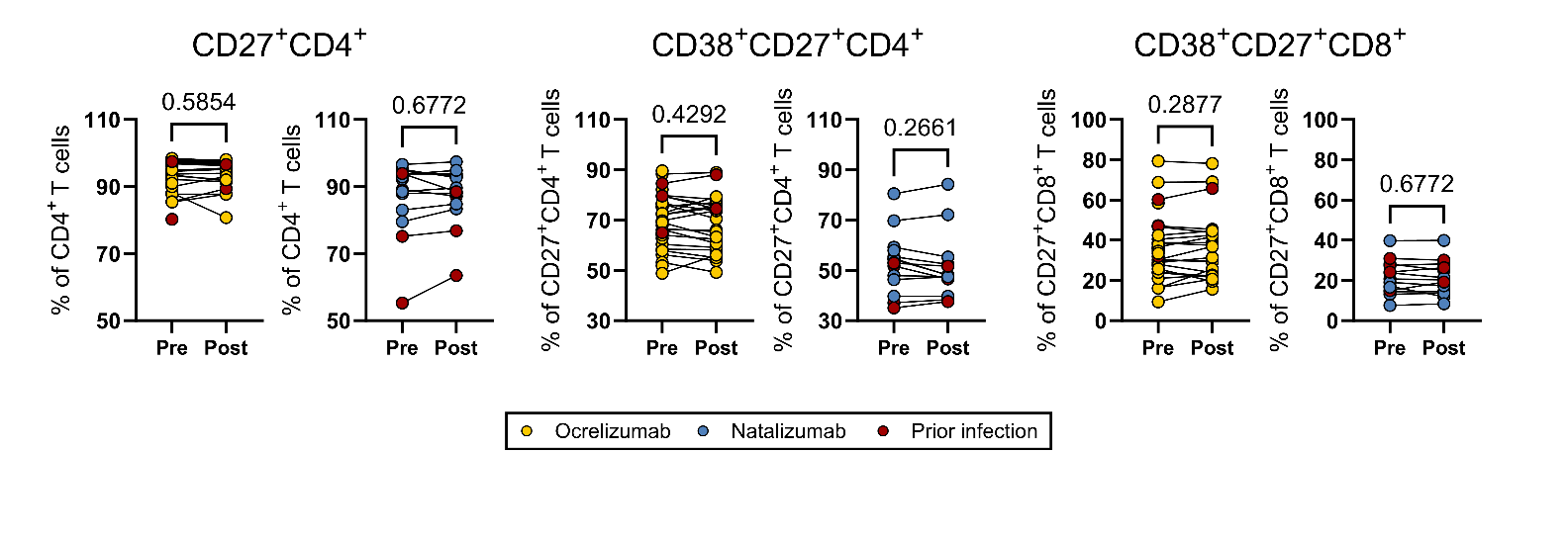
**

**Supplementary Figure 6.** Stability of selected immune populations across two time points in people with multiple sclerosis treated with ocrelizumab or natalizumab. **Statistical significance between time points by Wilcoxon-matched pairs signed rank test.**

**
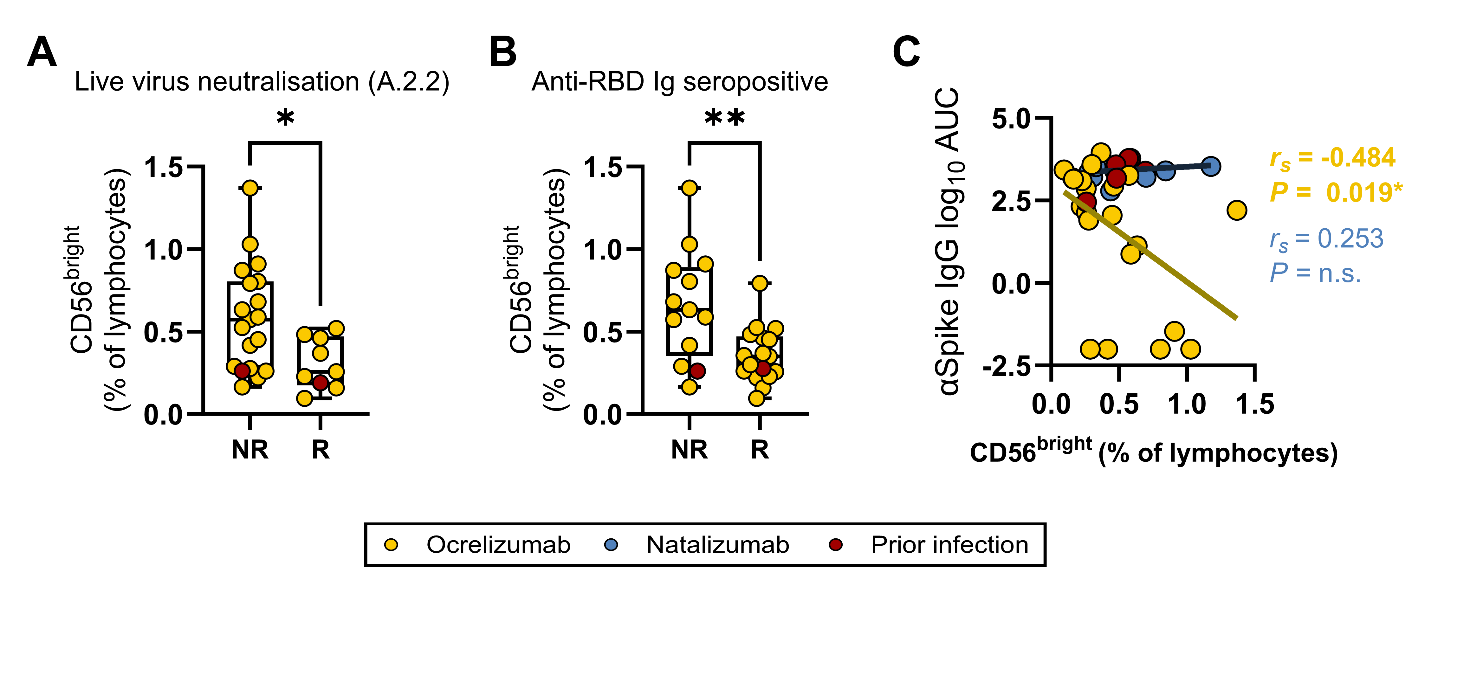
**

**Supplementary Figure 7. (A-B)** Pre-vaccination CD56^bright^ NK cells were elevated as a percentage of viable lymphocytes in people with multiple sclerosis on ocrelizumab who did not respond to SARS-CoV-2 booster vaccination as assessed by (A) effective neutralisation of live SARS-CoV-2 virus (A.2.2) and (B) seroconversion of anti-SARS-CoV-2 receptor binding domain Ig. **(C)** Pre-vaccination CD56^bright^ NK cells as a percentage of viable lymphocytes negatively correlated with anti-SARS-CoV-2 Spike IgG response titre in people with multiple sclerosis receiving ocrelizumab but not natalizumab. **Statistical significance between groups by Mann-Whitney and variable relationships by Spearman correlation. **P<0.01; *P<0.05; ns, non-significant.**
